# Green Biocatalysis of Xylitol Monoferulate: *Candida antarctica* Lipase B-Mediated Synthesis and Characterization of Novel Bifunctional Prodrug

**DOI:** 10.3390/biotech14020025

**Published:** 2025-04-02

**Authors:** Federico Zappaterra, Francesco Presini, Domenico Meola, Chaimae Chaibi, Simona Aprile, Lindomar Alberto Lerin, Pier Paolo Giovannini

**Affiliations:** Department of Chemical, Pharmaceutical and Agricultural Sciences, University of Ferrara, Via Luigi Borsari, 46, 44121 Ferrara, Italy

**Keywords:** ferulic acid, xylitol, green enzymatic synthesis, bifunctional prodrugs, biobased polyols, CaLB

## Abstract

Natural compounds with significant bioactive properties can be found in abundance within biomasses. Especially prominent for their anti-inflammatory, neuroprotective, antibacterial, and antioxidant activities are cinnamic acid derivatives (CAs). Ferulic acid (FA), a widely studied phenylpropanoid, exhibits a broad range of therapeutic and nutraceutical applications, demonstrating antidiabetic, anticancer, antimicrobial, and hepato- and neuroprotective activities. This research investigates the green enzymatic synthesis of innovative and potentially bifunctional prodrug derivatives of FA, designed to enhance solubility and stability profiles. Selective esterification was employed to conjugate FA with xylitol, a biobased polyol recognized for its bioactive antioxidant properties and safety profile. Furthermore, by exploiting *t*-amyl alcohol as a green solvent, the enzymatic synthesis of the derivative was optimized for reaction parameters including temperature, reaction time, enzyme concentration, and molar ratio. The synthesized derivative, xylitol monoferulate (XMF), represents a novel contribution to the literature. The comprehensive characterization of this compound was achieved using advanced spectroscopic methods, including ^1^H-NMR, ^13^C-NMR, COSY, HSQC, and HMBC. This study represents a significant advancement in the enzymatic synthesis of high-value biobased derivatives, demonstrating increased biological activities and setting the stage for future applications in green chemistry and the sustainable production of bioactive compounds.

## 1. Introduction

Oxidative stress is a metabolic condition of the body caused by an imbalance between the production and elimination of ROS (reactive oxygen species). ROS are constantly and physiologically produced by the body because of natural cellular metabolic processes [1,2]. These species naturally attack important biological molecules such as lipids and especially nucleic acids, generating peroxidized lipids in biological membranes and causing DNA damage, which can lead to mutations [3]. Consequently, if they are not effectively controlled by cellular mechanisms, they can result in chronic pathological conditions such as type II diabetes, neurodegenerative diseases including Alzheimer’s disease, hyperlipidosis, and cancers [2,4].

Natural compounds, such as ferulic acid (**1**) derived from cinnamic acid, have been reported as natural substances with anti-inflammatory, antibacterial, antiviral, and antioxidant activities [5]. These effects are attributed to their molecular structure, characterized by a phenoxy group and a hydroxyl group that donate electrons to free radicals, neutralizing their effects and forming a quinonic intermediate excreted through bile [6,7]. Furthermore, the phenolic ring inherent to the compound’s structure confers a range of antibacterial [8], anti-inflammatory [9], and antiviral properties [10] due to its modulation of enzymatic activity [11], signal transduction [12], and gene expression [6].

Ferulic acid is a phenolic compound derived from hydrocinnamic acid that naturally occurs in plant cell walls, esterified to hemicellulose [13]. It is found in wheat and other cereals [6]. This compound exhibits a broad spectrum of therapeutic effects against chronic diseases such as cancer [14], diabetes [15], cardiovascular diseases [16], and neurodegenerative disorders [17], largely due to its pronounced antioxidant activity. It is a powerful antioxidant for cellular membranes [18], efficiently scavenging free radicals such as the superoxide anion radical [19] and thereby inhibiting lipid peroxidation [6,20].

Cinnamic acid derivatives such as ferulic acid therefore hold significant potential, which is, however, limited by their poor water solubility [21] and, consequently, low bioavailability [22]. Thus, the synthesis of a ferulic acid derivative via esterification with xylitol, a polyol widely used industrially as a low-calorie natural sweetener that is highly soluble in aqueous solvents, was proposed [23]. Recent studies have demonstrated that xylitol and other polyalcohols represent a superior alternative to sugars such as sucrose, with markedly positive results in diabetes [24,25]. Recently, xylitol has been reported as a polyol with anti-inflammatory [26], antiviral [27], and antibacterial properties [27], mainly due to its alkalizing power, which creates an environment highly unfavorable for bacterial growth [24,28]. Xylitol is a bio-based polyol derived from xylose, which can be produced sustainably from lignocellulosic biomasses through both chemical and enzymatic processes. The valorization of non-edible biomasses into fermentable sugars, including xylose, has gained increasing attention as a green alternative to petroleum-derived feedstocks, aligning with the principles of circular bioeconomy and sustainable chemistry. Recent studies have demonstrated the efficient conversion of biomass-derived polysaccharides into xylose and other valuable sugar derivatives, offering promising routes for xylitol production. For example, waste wheat bran, a byproduct of the food industry, has been directly hydrolyzed via both enzymatic and microwave-assisted chemical routes to yield fermentable sugars, which are subsequently converted into high-value fine chemicals, such as carotenoids and lipids, demonstrating a cascade approach for biomass valorization [29]. Similarly, a high-pressure CO_2_-based catalytic pretreatment has been optimized to achieve selective hydrolysis of hemicellulose into xylose and xylo-oligosaccharides (XOS), enabling the efficient downstream enzymatic hydrolysis of biomass residues for glucose production [30]. These advancements highlight the potential of tailored and green approaches for the sustainable production of xylose, reinforcing xylitol’s bio-based origin and its role in environmentally friendly bioprocesses.

The synthesis of prodrugs that follow green protocols can be designed through enzyme-catalyzed esterification [31]. Enzymatic catalysis offers advantages over traditional chemical catalysis because enzymes ensure reaction specificity, reducing the risk of by-product formation and, above all, avoiding toxic products [32]. Furthermore, when immobilized on a solid matrix, enzymes can be reused. Lipases are the most used enzymes in esterification [33]. Biologically, they catalyze the formation of glycerol esters [34], but in low-water environments, they can also catalyze the formation of various types of esters [35]. Lipase activity depends on several parameters, such as temperature, pH, and the type of solvent [36]. Lipases are often used in esterification due to their stereo- and regio-selectivity, particularly with polyols, on the primary hydroxyl group [37]. Additionally, CaLB exhibits high stability over a wide range of temperatures and, like all lipases, does not require cofactors [38,39].

**Scheme 1:**
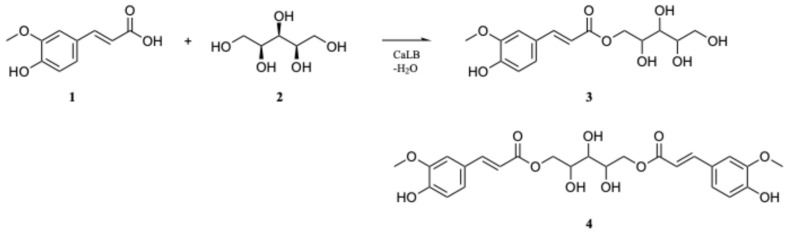
A biocatalyzed esterification reaction for the selective synthesis of xylitol monoferulate (XMF, **3**) from ferulic acid (FA, **1**) and xylitol (**2**), with traces of xylitol bis(ferulate) (**4**) as a byproduct.

The aim of this scientific work is the design of selective esterification protocols for the production of a potential prodrug, xylitol monoferulate (**3**), using biocatalytic approaches (Scheme 1). The resulting derivative is expected to exhibit increased solubility in aqueous solvents and, consequently, enhanced bioavailability, as well as improved antioxidant activity. To achieve the esterification of cinnamic acid derivatives (CAs), reaction parameters such as temperature, reaction time, enzyme concentration, and water content were investigated. The selection of an appropriate solvent plays a crucial role in enzymatic reactions, particularly when dealing with substrates exhibiting highly contrasting solubility profiles. In this study, *t*-amyl alcohol was chosen as the reaction medium due to its ability to maintain a monophasic system while effectively solubilizing both ferulic acid (FA, **1**), a poorly water-soluble bioactive compound, and xylitol (**2**), a highly hydrophilic polyol. Unlike other common organic solvents, which resulted in biphasic systems or poor dissolution of one of the reagents, *t*-amyl alcohol provided a homogeneous environment, enabling efficient enzymatic esterification. Furthermore, *t*-amyl alcohol is classified as a green solvent [40] as it exhibits relatively low toxicity, high biodegradability, and reduced environmental impact compared to traditional organic solvents such as THF or acetonitrile. Its compatibility with biocatalytic processes, combined with its ability to support an efficient reaction system, makes it a sustainable and practical choice for this study.

To the best of our knowledge, no previous work has proposed the enzymatic esterification of ferulic acid (FA) and xylitol, starting from two solid substrates with such markedly different polarities. Furthermore, the enzymatic synthesis of the ferulic acid–xylitol ester as a potential prodrug has not been previously reported. Typically, comparable enzymatic esterification strategies involve the use of one of the two substrates in its liquid form, allowing it to act both as a reagent and a solvent, as is commonly observed in reactions utilizing liquid alcohols. Indeed, several previous studies have explored enzymatic esterification strategies involving ferulic acid and structurally related cinnamic acid derivatives. However, in contrast to our work, these studies typically rely on the use of liquid alcohol as one of the starting reagents, which also acts as a co-solvent, ensuring better solubility and reaction homogeneity. This approach simplifies mass transfer limitations and improves enzymatic efficiency by avoiding phase separation issues. For example, Lerin et al. reported the enzymatic synthesis of a ferulic acid–geraniol ester, a potential prodrug designed to enhance bioavailability and therapeutic efficacy [41]. In their system, geraniol, a monoterpenoid alcohol, was liquid at reaction conditions, acting as both a reagent and a solvent. This dual role facilitated enzymatic esterification, improving conversion yields while avoiding the solubility challenges posed by solid polyols like xylitol. Similarly, Cabral do Nascimento et al. [42] investigated the enzymatic esterification of cinnamic acid derivatives with geraniol, demonstrating the viability of using liquid alcohol as a reactant and reaction medium. Their approach optimized reaction conditions through the design of experiments (DOE) and artificial neural networks (ANNs), reinforcing the idea that phase homogeneity significantly impacts enzymatic efficiency. Other studies have primarily focused on small alkyl esters of ferulic acid, such as methyl and ethyl ferulates, rather than complex polyol esters. For instance, Kong et al. [43] demonstrated that methyl and ethyl ferulates exhibit enhanced antifungal activity compared to free ferulic acid. However, their synthesis follows a conventional chemical route rather than an enzymatic approach. Similarly, Wang et al. [44] synthesized a series of ethyl esters of ferulic acid, focusing on their potential as xanthine oxidase inhibitors, further confirming the bioactivity enhancement achieved through esterification. Finally, the comprehensive review by Li et al. [NO_PRINTED_FORM]extensively discusses the pharmacology, pharmacokinetics, and derivatives of ferulic acid, highlighting various esterified forms and their therapeutic applications. However, most reported ferulic acid esters involve small alcohols or fatty acid esters, rather than polyols, emphasizing the novelty of our enzymatic approach in synthesizing a water-soluble xylitol monoester for potential pharmaceutical applications. The major distinction between our work and these previous studies lies in the use of two solid reagents (ferulic acid and xylitol), each with significantly different polarities, requiring an innovative reaction system to achieve homogeneous enzymatic esterification. Unlike geraniol-based or short-chain alkyl ester syntheses, our method does not benefit from a liquid alcohol phase, necessitating a solvent system that can simultaneously dissolve both reactants while maintaining enzymatic activity. This highlights the unique challenges and advancements introduced in our work, further emphasizing the importance of biocatalytic strategies for prodrug synthesis.

## 2. Materials and Methods

### 2.1. Material

Lipozyme 435 (immobilized *Candida antarctica* lipase B) was purchased from Novozymes A/S (Fredericksberg, Denmark). Ferulic acid (purity > 98%), xylitol (purity > 98%), and silica gel (60°, 70–230 mesh, 63–200 µL) were purchased from Sigma-Aldrich (Buchs, Switzerland). 2-Methyl-2-butanol (*t*-amyl alcohol, or 2M2B) and all other solvents were ACS grade, were purchased from Sigma-Aldrich (Buchs, Switzerland), and were used without further purification. TLC plates (Silica Gel 60, 5 × 10 cm) were purchased from Merck (Berlin, Germany). Molecular sieves (3Å, beads, ≥97%) were purchased from Merck (Darmstadt, Germany). NMR spectra were recorded using a Varian 400 MHz spectrometer (Varian, Palo Alto, CA, USA).

### 2.2. Biocatalytic Synthesis of Xylitol Monoferulate (XMF, ***3***)

The reactions were carried out in 10 mL screw-capped flasks placed in an oil bath with magnetic stirring. The effect of reaction variables on the conversion of the acid to monoester was studied, starting from an initial reaction with a molar ratio (of **1** to **2**) of 1:1. Subsequently, the effects of different molar ratios, such as 1:3, 1:5, 3:1, and 5:1; the temperature (from 50 to 90 °C); the biocatalyst amount (20 to 100 g/L); the molecular sieves amount (0 or 100 mg); and the reaction solvent volume (*t*-amyl alcohol, 2 or 5 mL) were evaluated. The reactions, kept at constant temperature and stirring, were monitored via ¹H-NMR spectroscopic analysis (24 h, 48 h, 72 h, up to 192 h). Under the initial reaction conditions (1:1 molar ratio, 50 °C), traces of diester 4 were detected, which was subsequently separated by flash column chromatography and characterized via NMR. All reactions were performed in triplicate, and the reported conversion values represent the mean of three independent experiments, with the error expressed as the standard deviation (SD).

To ensure reproducibility and facilitate comparison with other studies, the amount of immobilized enzyme was expressed both as the concentration (100 g/L) and as the weight-on-weight (*w*/*w*) relative to the limiting reagent. Based on the optimized reaction conditions, where ferulic acid was used at 258 mM in t-amyl alcohol (2 mL), the corresponding masses of ferulic acid, xylitol (limiting reagent), and immobilized enzyme were 0.100 g, 0.0157 g, and 0.200 g, respectively. This resulted in an enzyme-to-xylitol ratio of 12.74 *w*/*w*, further highlighting the stability and efficiency of the immobilized biocatalyst in this system (Table 1).

### 2.3. Thin-Layer Chromatography (TLC)

TLC analysis was employed to monitor the progress of the reactions. For each reaction, 50 µL of the reaction medium was sampled, diluted in 1 mL of methanol (MeOH), and analyzed on TLC plates. A mobile phase composed of ethyl acetate/acetonitrile/acetic acid (55:45:3, *v*/*v*/*v*) was used for the separation of the monoester, while an eluent phase of ethyl acetate/hexane (1:1, *v*/*v*) was employed for the separation and characterization of the diester byproduct. The products were detected under UV light (λ = 254 nm).

### 2.4. Purification and Spectroscopic Characterization of Esters

Flash column chromatography with silica gel was used to purify the esters synthesized during the reactions. An ethyl acetate/hexane eluent mixture (1:1, *v*/*v*) was employed to isolate the diester byproduct (**4**) while the eluent mixture for isolating xylitol monoferulate (**3**) consisted of ethyl acetate/acetonitrile/acetic acid (55:45:3, *v*/*v*/*v*). Under non-optimized reaction conditions (1:1 molar ratio, 50 °C), traces of a diester by-product, xylitol bis(ferulate), were detected and subsequently separated via flash column chromatography and characterized by NMR spectroscopy. However, its formation was negligible under optimized conditions, suggesting that CaLB exhibits strong selectivity for monoester formation over polyacylation. This selectivity may be attributed to a lower affinity of the monoester to the enzyme’s active site compared to the initial substrates, preventing its further acylation. The presence of multiple hydroxyl groups in xylitol likely plays a role in enzyme–substrate interactions, potentially stabilizing the monoester in solution and preventing re-entry into the catalytic pocket.

Future studies could employ molecular dynamics (MDs) simulations and molecular docking analyses to investigate substrate–enzyme interactions, as well as kinetic and thermodynamic profiling to validate the preferential monoesterification pathway. These findings reinforce the advantage of enzymatic catalysis in selectively producing mono-functionalized derivatives which would be difficult to achieve using traditional chemical esterification routes. The collected fractions were analyzed using TLC with the same eluent used in the flash column. Fractions containing the product were combined in a flask; the solvent evaporated using a rotary evaporator (rotavapor), and the residue was characterized via NMR spectroscopy.

### 2.5. NMR Spectroscopy

NMR spectra were recorded using a Varian 400 MHz spectrometer (Varian, Palo Alto, CA, USA). ^1^H-NMR and ^13^C-NMR spectra were acquired at operating frequencies of 400 MHz and 100 MHz, respectively. Chemical shifts (δ) are reported in parts per million (ppm) relative to the internal standard tetramethylsilane (TMS, δ = 0.00 ppm) or residual solvent signals. Two-dimensional NMR experiments, including COSY (Correlation Spectroscopy), HSQC (Heteronuclear Single Quantum Coherence), and HMBC (Heteronuclear Multiple Bond Correlation), were performed using standard pulse sequences to confirm structural assignments. Spectra were processed and analyzed using the MestReNova software v14.2.3 (Mestrelab Research, Santiago de Compostela, Spain). To determine the conversion of (**1)** into (**3**), the ratio between the area under the ester peak (**3**) (δ = 6.4 ppm) and the sum of the areas under the ester peak (**3**) (δ = 6.4 ppm) and the acid peak (**1**) (δ = 6.3 ppm) was calculated and multiplied by 100. This value represents the relative conversion percentage. When (**1**) is the limiting reagent, the relative conversion corresponds to the yield. However, when (**2**) (the alcohol) is the limiting reagent, the molar fraction of (**3**), deduced from the NMR, must be compared to the initial molar fraction of (**2**) in the mixture to calculate the actual yield. The analysis of the products using ^1^H-, ^13^C-, COSY, HSQC, and HMBC-NMR spectroscopy enabled the characterization of product (**3**) (see the Supplementary Materials), and byproduct (**4**). During the experimental development phase, ¹H-NMR analysis was also employed to monitor the reaction progress every 24 h. From each reaction, 50 µL samples were taken, dried using a rotary evaporator to remove the reaction solvent, and subsequently diluted in 800 µL of deuterated methanol (CD_3_OD). Xylitol monoferulate (**1**): ^1^H NMR (400 MHz, CD_3_OD) δ 7.65 (d, *J* = 15.9 Hz, 1H), 7.18 (d, *J* = 2.0 Hz, 1H), 7.07 (dd, *J* = 8.2, 2.0 Hz, 1H), 6.81 (d, *J* = 8.2 Hz, 1H), 6.38 (d, *J* = 15.9 Hz, 1H), 4.52–4.18 (m, 2H), 3.89 (s, 3H), 3.86–3.54 (m, 5H). ^13^C NMR (101 MHz, CD_3_OD) δ 167.78, 149.24, 147.96, 145.60, 126.33, 122.69, 115.07, 113.93, 110.30, 72.34, 70.65, 70.11, 65.43, 62.76, 55.01.

## 3. Results and Discussion

### 3.1. The Effect of Temperature on the Biocatalyzed Synthesis of Xylitol Monoferulate

**Figure 1:**
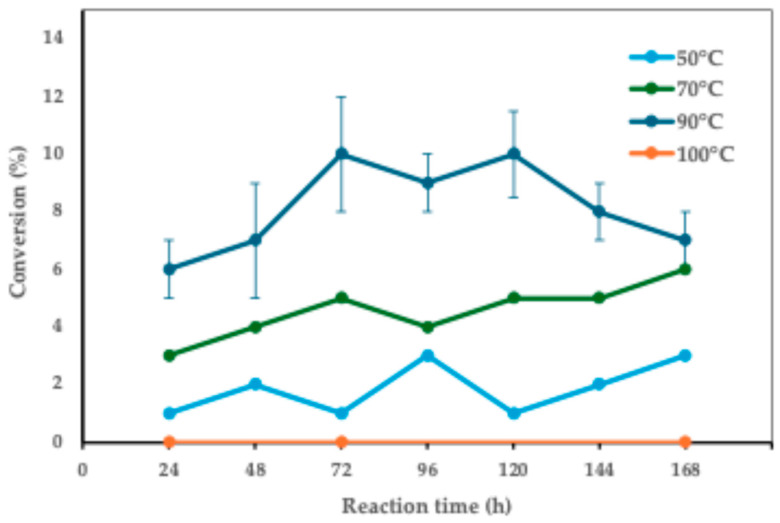
The effect of temperature on the conversion of **1** to **3**. Reaction conditions: 1:1 molar ratio (FA:xylitol), 10 g/L CaLB, 5 mL *t*-amyl alcohol, magnetic stirring at 180 rpm. Ferulic acid (FA): 0.8 mmol (0.155 g), xylitol: 0.8 mmol (0.122 g), enzyme-to-limiting reagent ratio: 0.00041:1 *w*/*w*.

Figure 1 shows the results of the study on the effect of temperature variation on the final reaction yield.

Starting from the initial reaction with a 1:1 acid/alcohol molar ratio and an enzyme concentration of 10 g/L, various temperatures were tested, and the results are shown in Figure 1, which reports the percentage conversion of xylitol ferulate as a function of time. As observed in the graph in Figure 1, there is an increase in the percentage of conversion correlating with the rise in temperature from 50 °C to 70 °C and 90 °C due to the increased enzymatic activity as the optimal temperature is approached. Maximum conversions are observed at 72 h. During this initial phase, a conversion of 10% is obtained at 90 °C, 5% at 70 °C, and finally, 1% at 50 °C. At 100 °C, no product formation is observed, likely due to enzyme denaturation and the consequent loss of enzymatic activity at this temperature. Based on these findings, a temperature of 90 °C was selected for subsequent steps. *Candida antarctica* lipase B (CaLB) is well known for its exceptional thermostability and solvent tolerance [45]. The previous literature reported that CaLB catalytic is able to resist even at temperatures exceeding 100 °C when water is limited. For example, Rejasse et al. observed that free CaLB remained active up to 110 °C in a nearly anhydrous (solvent-free) system, showing identical conversion yields at that high temperature as under conventional conditions [46]. Likewise, immobilized CaLB preparations have withstood exposure to boiling organic solvents (e.g., in dimethylformamide at 153 °C) with minimal loss of activity [47]. Such resilience is attributed to the protective effect of non-aqueous media on the enzyme structure: in the absence of bulk water, enzymes become markedly more thermostable, with dehydrated lipases reported to maintain high activity at 100 °C for hours without denaturation [48]. In this context, the use of an organic solvent like *t*-amyl alcohol is particularly advantageous. This solvent’s high boiling point (≈102 °C) permits reactions at or above 100 °C without evaporation, and its non-aqueous environment helps preserve the enzyme’s conformation and function at elevated temperatures [35]. Consequently, CaLB can operate under relatively harsh reaction conditions (high temperatures in *tert*-amyl alcohol) while retaining robust enzymatic performance, as observed in the present system.

The selection of 90 °C as the optimal temperature for this enzymatic esterification is strongly supported by literature reports on *Candida antarctica* lipase B (CaLB), which is widely recognized for its exceptional thermostability compared to other lipases. Unlike mammalian or microbial lipases that typically exhibit optimal activity at around 30–50 °C, CaLB retains significant catalytic efficiency, even at temperatures above 90 °C and particularly in non-aqueous media. Previous studies, including our own work on the enzymatic esterification of ibuprofen and cinnamic acid derivatives, have demonstrated that CaLB remains highly active under these conditions, particularly in t-amyl alcohol, where near-quantitative conversions (>95%) were achieved at 90 °C [20,28,33]. Furthermore, other reports have documented CaLB activity at even higher temperatures in organic solvents, further validating the choice of 90 °C as a suitable operating temperature for this system.

The reaction timeframe observed in this study is consistent with the nature of enzymatic esterification, which generally operates under different kinetic constraints compared to classical Fischer esterifications or other chemical routes. While chemical esterifications often rely on strong acid catalysts, temperatures exceeding 120 °C, elevated pressures, or inert atmospheres, the biocatalytic approach prioritizes selectivity, environmental compatibility, and product purity over speed. A major advantage of this enzymatic route is the ability to selectively esterify the primary hydroxyl group of xylitol, eliminating the need for multiple protection and deprotection steps required in traditional chemical synthesis. Additionally, the biocatalyst (CaLB) is biodegradable, non-toxic, and reusable, further reinforcing the sustainability of the process.

The observed differences in reaction kinetics, including both linear and sigmoidal trends, can be attributed to multiple interconnected factors. Linear conversion profiles suggest steady-state enzyme kinetics, where CaLB operates under relatively stable conditions without significant substrate depletion or loss of catalytic efficiency. In contrast, sigmoidal kinetics imply the presence of an initial lag phase, likely caused by substrate dissolution limitations, diffusion constraints, or enzyme activation phenomena in the early stages of the reaction. Given that ferulic acid and xylitol exhibit markedly different solubility behaviors in *t*-amyl alcohol, initial mass transfer effects may play a role in delaying the establishment of a fully reactive system. Additionally, the potential for enzyme structural adjustments within the solvent environment could contribute to the delayed onset of maximum catalytic activity.

While CaLB is widely recognized for its robustness, long-term exposure to organic solvents can induce gradual conformational changes, aggregation effects, or partial deactivation. However, under the experimental conditions employed in this study, no significant enzyme deactivation was observed, as evidenced by the sustained catalytic activity and high conversion rates achieved over extended reaction times. These findings suggest that CaLB remains catalytically competent throughout the reaction, even in a hydrophobic solvent system.

It is important to emphasize that the focus of this study was not to perform an exhaustive kinetic analysis of CaLB but rather to develop an optimized enzymatic esterification protocol for the selective production of xylitol monoferulate as a potential prodrug. The reaction was designed to maximize conversion efficiency while ensuring selective monoester formation, with an emphasis on improving water solubility and potential therapeutic applications. Future studies dedicated to the mechanistic investigation of enzyme behavior in this system, including enzyme deactivation kinetics and mass transfer limitations, could provide further insights into the underlying catalytic phenomena.

To assess the stability and potential leaching of the immobilized enzyme during the reaction, a test was performed where the biocatalyst was removed after 24 h, and the reaction was allowed to proceed further in the absence of the immobilized lipase. No additional product formation was detected, indicating that the enzyme did not leach into the reaction medium and confirming that the observed conversion was entirely dependent on the immobilized form. Additionally, to evaluate the impact of solvent conditions on enzyme integrity, control reactions were conducted using free (non-immobilized) CaLB. Under identical conditions, the enzyme visibly denatured and precipitated upon introduction into the reaction medium, and no product formation was observed at any reaction time or temperature. These findings demonstrate that immobilization provides a robust and stable biocatalytic system under the optimized conditions, preventing enzyme leaching and degradation.

### 3.2. The Effect of Molar Ratio on the Conversion of ***1*** to ***3***

**Figure 2:**
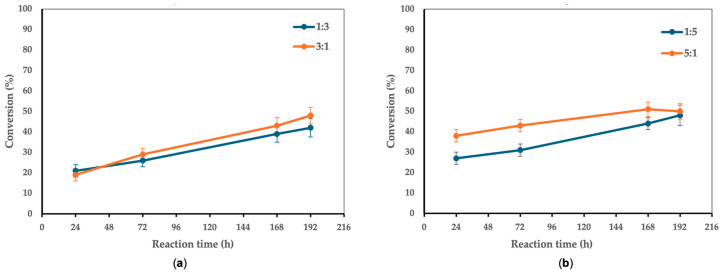
The effect of the acid-to-alcohol molar ratio on the conversion of **1** to **3**. Reaction conditions: (**a**) molar ratio 1:3 vs. 3:1, 10 g/L CaLB, 5 mL *t*-amyl alcohol, 90 °C, magnetic stirring at 180 rpm; (**b**) molar ratio 1:5 vs. 5:1, 10 g/L CaLB, 5 mL *t*-amyl alcohol, 90 °C, magnetic stirring at 180 rpm. For the 1:3 molar ratio, ferulic acid (FA) was used at 0.8 mmol (0.155 g), while xylitol was 2.4 mmol (0.365 g), with an enzyme-to-limiting reagent ratio of 0.000137:1 *w*/*w*. For the 3:1 molar ratio, FA was 0.8 mmol (0.155 g), while xylitol was 0.267 mmol (0.041 g), with an enzyme-to-limiting reagent ratio of 0.001232:1 *w*/*w*. When the 1:5 ratio was tested, FA was 0.8 mmol (0.155 g), while xylitol was 4.0 mmol (0.609 g), with an enzyme-to-limiting reagent ratio of 0.000082:1 *w*/*w*.

Figure 2 shows the effects of varying the acid-to-alcohol molar ratio on the progress of the reaction.

Once the optimal temperature was established, the effect of varying the acid-to-alcohol molar ratio on the ester conversion percentage was studied, with the results plotted as a function of time. Specifically, molar ratios (acid:alcohol) of 1:3 and 3:1, as well as 1:5 and 5:1, were tested. From the comparison of the obtained curves, better conversion was observed with an excess of acid rather than alcohol. For example, at 168 h, the molar ratios 1:3 and 3:1 resulted in conversions of 39% and 43%, respectively. Similarly, the molar ratios 1:5 and 5:1 showed conversions of 44% and 51% at 168 h. Therefore, the 5:1 molar ratio proved to be optimal for the conversion of acid into monoester at the reaction temperature of 90 °C. This result could be attributed to the effect of the polyhydroxylic nature of xylitol on the enzymatic conformation. Polyhydric alcohols, including xylitol and sorbitol, have been shown to influence enzyme catalysis in a concentration-dependent manner [49,50]. At moderate levels, these polyols can stabilize enzyme conformation by forming hydrogen bonds with the protein surface, thereby preserving structural integrity and catalytic function. However, at higher concentrations, they may exert an inhibitory effect by altering the hydration shell of the enzyme, increasing viscosity, and reducing substrate diffusion [51,52,53]. In the case of *Candida antarctica* lipase B (CaLB), polyols such as xylitol can engage in extensive hydrogen bonding with key structural regions of the enzyme, potentially interfering with its active site accessibility and conformational flexibility [54]. This phenomenon is particularly relevant in esterification reactions, where the presence of multiple hydroxyl groups on xylitol may affect the enzyme’s conformation, thereby hindering substrate turnover and slowing down the overall reaction [55]. Additionally, polyols have a high affinity for water, which can increase localized water activity and promote ester hydrolysis, thereby reducing product yield [36]. Conversely, an excess of acid mitigates these inhibitory effects by shifting the reaction equilibrium toward ester formation while simultaneously maintaining a more hydrophobic environment around the enzyme. This hydrophobic microenvironment is known to favor lipase activity by stabilizing the active conformation and minimizing interference from hydrogen-bonding interactions with the polyol substrate [56,57]. The observed selectivity for monoester formation over diesterification may also be attributed to a lower affinity of the monoester for the enzyme’s active site, leading to its preferential release into the solvent phase rather than further acylation [58,59,60]. These findings are consistent with literature reports that demonstrate that polyhydric alcohols can function as both enzyme stabilizers and inhibitors, depending on their concentration, solvent environment, and the nature of the biocatalyst. Thus, careful optimization of substrate ratios is critical to ensuring high reaction efficiency and selectivity in enzymatic esterifications involving polyols.

### 3.3. The Effect of CaLB Concentration on the Conversion of ***1*** to ***3***

**Figure 3:**
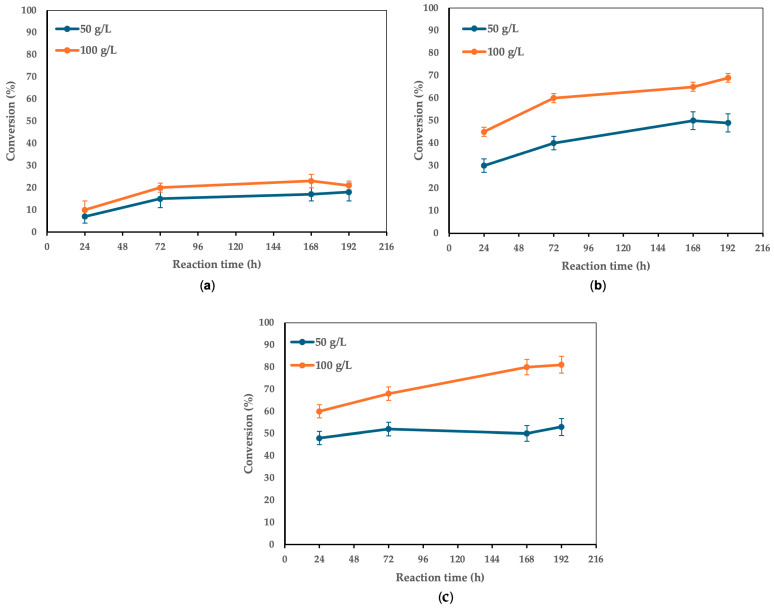
The effect of enzyme concentration on the conversion of 1 to 3. Reaction conditions: (**a**) molar ratio 1:1, 50 vs. 100 g/L CaLB, 5 mL *t*-amyl alcohol, 90 °C, magnetic stirring at 180 rpm; (**b**) molar ratio 3:1, 50 vs. 100 g/L CaLB, 5 mL *t*-amyl alcohol, 90 °C, magnetic stirring at 180 rpm; (**c**) molar ratio 5:1, 50 vs. 100 g/L CaLB, 5 mL *t*-amyl alcohol, 90 °C, magnetic stirring at 180 rpm. For the 1:1 molar ratio, FA was 0.8 mmol (0.155 g), while xylitol was 0.8 mmol (0.122 g). The enzyme-to-limiting reagent ratio was 0.0021:1 *w*/*w* at 50 g/L CaLB and 0.0041:1 *w*/*w* at 100 g/L CaLB. For the 3:1 molar ratio, FA was 0.8 mmol (0.155 g), while xylitol was 0.267 mmol (0.041 g). The enzyme-to-limiting reagent ratio was 0.0062:1 *w*/*w* at 50 g/L CaLB and 0.0123:1 *w*/*w* at 100 g/L CaLB. For the 5:1 molar ratio, FA was 4.0 mmol (0.776 g), while xylitol was 0.160 mmol (0.024 g). The enzyme-to-limiting reagent ratio was 0.0103:1 *w*/*w* at 50 g/L CaLB and 0.0205:1 *w*/*w* at 100 g/L CaLB.

Subsequently, the effect of increasing enzyme concentration at its optimal temperature (90 °C) was tested on different molar ratios, considering only the ratios with an excess of acid over alcohol (Figure 3).

Enzyme concentrations of 50 g/L and 100 g/L were tested with acid/alcohol molar ratios of 1:1, 3:1, and 5:1, and the results are presented in Figure 3a–c as a function of time. From an initial comparison of the conversion percentages, better results were obtained with a molar ratio of 5:1 at the same enzyme concentration (50 or 100 g/L). Specifically, at 168 h and 50 g/L of the enzyme, conversions of 17% for a 1:1 molar ratio, 49% for 3:1, and 50% for 5:1 were observed. Similarly, at 168 h and 100 g/L of the enzyme, conversions of 23% for the 1:1 ratio, 65% for 3:1, and 80% for 5:1 were recorded.

Thus, an improvement in yield was consistently observed with an increase in the acid/alcohol molar ratio. As seen from the comparison of the curves corresponding to 100 g/L, the combination of the previously optimized molar ratio parameter with the maximum enzyme concentration (100 g/L) provided the best conversion results. This improvement is likely due to the fact that increasing the enzyme concentration increases the number of active sites available for catalysis relative to the substrate concentration. From this analysis, the optimized esterification parameters determined at this stage are as follows: a reaction temperature of 90 °C for optimal enzymatic biocatalytic activity, an acid/alcohol molar ratio of 5:1, and an enzyme concentration of 100 g/L.

### 3.4. The Effect of Ferulic Acid (***1***) and Xylitol (***2***) Concentration on the Conversion of ***1*** to ***3***

In all the previous experiments, the reaction solvent volume (*t*-amyl alcohol) was kept constant at 5 mL. An additional test was then conducted to evaluate the effect of concentration on the reaction yield. The volume of *t*-amyl alcohol was reduced from 5 mL to 2 mL (resulting in a change in the ferulic acid (**1**) concentration from 103 mM to 258 mM). It is important to note that the enzyme concentration was maintained at 100 g/L, as previously optimized, ensuring that any observed effects were due solely to changes in reagent concentration and not to variations in biocatalyst loading.

**Figure 4:**
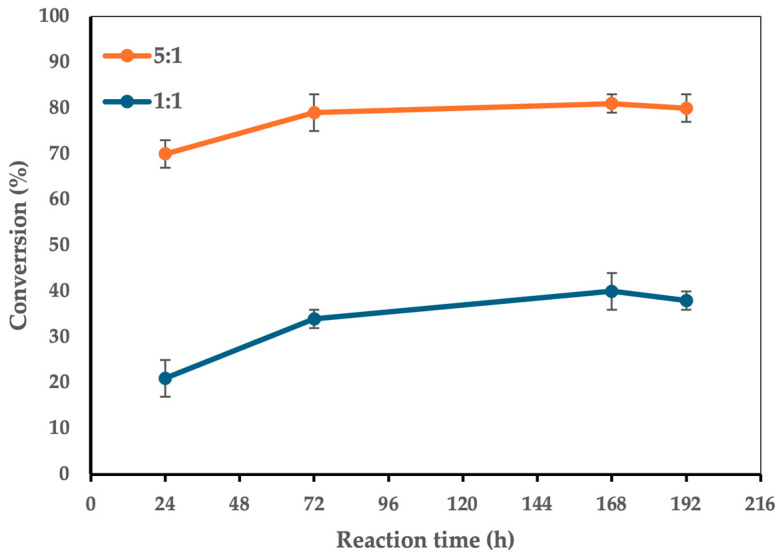
The effect of reaction concentration on the conversion of **1** to **3**. Reaction conditions: molar ratio 5:1, 100 g/L CaLB, 90 °C, magnetic stirring at 180 rpm, reaction volume 2 mL vs. 5 mL. For the 2 mL reaction volume, FA was 0.516 mmol (0.100 g), while xylitol was 0.103 mmol (0.016 g). The enzyme-to-limiting reagent ratio was 12.74:1 *w*/*w*.

From the results plotted as a function of time (Figure 4), a positive correlation between the increase in reaction concentration and the conversion percentage of the acid to the monoester can be observed.

This result is attributed to the fact that a smaller reaction volume, and thus an increased reaction concentration, enhances mass transfer between the reagents, allowing them to interact more effectively with the enzyme’s active site. Consequently, the esterification yield is improved. To further evaluate the feasibility of this enzymatic esterification process, we estimated its productivity in terms of product formation per unit volume and time. Based on the optimized reaction conditions, where xylitol was the limiting reagent (initial concentration 51.6 mM) and a quantitative conversion was achieved in 72 h, the productivity was calculated as 0.7 mmol/L/h, corresponding to 243 mg/L/h. While this value reflects the inherently slow kinetics of biocatalyzed esterifications, it remains comparable to other enzymatic processes reported in the literature for the synthesis of structured lipids and other esters. Future work aimed at process intensification—such as enzyme immobilization strategies, higher substrate loadings, or reaction engineering approaches—could further improve productivity and enhance large-scale applicability.

### 3.5. The Effect of Water on the Conversion of ***1*** to ***3***

**Figure 5:**
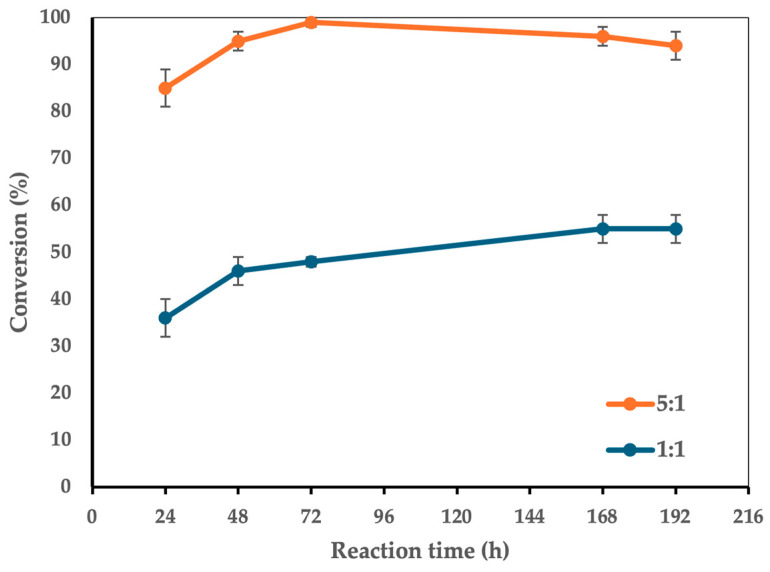
The effect of dehydration on the conversion of **1** to **3**. Reaction conditions: molar ratio 1:1 vs. 5:1, 100 g/L CaLB, 90 °C, 5 mL *t*-amyl alcohol, 100 mg molecular sieves (3Å pore size), magnetic stirring at 180 rpm. For the 1:1 molar ratio, FA was 0.515 mmol (0.100 g), while xylitol was 0.515 mmol (0.078 g). The enzyme-to-limiting reagent ratio was 6.38:1 *w*/*w*. For the 5:1 molar ratio, FA was 0.515 mmol (0.100 g), while xylitol was 2.575 mmol (0.392 g). The enzyme-to-limiting reagent ratio was 5.00:1 *w*/*w*.

The final test conducted in this study evaluated the effect of dehydration on the conversion of **1** to **3**. Instead of evaluating the effect of different concentrations of molecular sieves, this study focused on assessing the impact of controlled dehydration by introducing a specific number of molecular sieves sufficient to remove only the water produced as a co-product of the enzymatic reaction (Figure 5).

The number of 3Å molecular sieves was used based on their ability to remove water molecules, which is 20% of their weight (with 100 mg of sieves, all potential water molecules generated as a byproduct of the direct esterification process were removed). A comparison was made between conditions with no sieves and those with 100 mg of sieves, revealing a significant improvement in monoester yield due to water removal, which drives the reaction toward the products. The addition of 100 mg of molecular sieves at a reaction temperature of 90 °C resulted in a significant increase in conversion, reaching nearly >98 ± 2% at 72 h under the optimal molar ratio of 5:1 between the reagents. In contrast, under a 1:1 molar ratio, conversion reached 48% at 72 h—still a very high yield considering the non-optimal molar ratio for conversion. While this study primarily focuses on the optimized biocatalytic synthesis of xylitol monoferulate, future work will aim to assess its solubility and stability profiles. Previous reports on similar polyol esters, including those of glycerol and erythritol, have demonstrated significant improvements in aqueous solubility [20,33]. Given the higher intrinsic hydrophilicity of xylitol, we anticipate that its ferulate ester may similarly exhibit enhanced solubility, making this enzymatic approach a promising strategy for potential prodrug development [61,62]. In the initial reaction conditions (1:1 molar ratio, prolonged reaction times), traces of a by-product, xylitol bis(ferulate), were detected. However, its formation was negligible under optimized conditions, where selectivity toward the monoester was maximized. The minor presence of this by-product indicates that the reaction conditions effectively direct esterification toward the desired xylitol monoferulate. Due to the by-product’s low concentration and its challenging separation from ferulic acid (similar retention times), its quantitative monitoring was not feasible. Through process optimization, the reaction time was significantly reduced compared to the initial screening conditions while still achieving high conversion efficiencies well before the original 8-day timeframe. Although enzymatic reactions may appear kinetically slower than purely chemical alternatives, they provide key advantages in terms of selectivity, environmental impact, and energy efficiency, all of which align with green chemistry principles [63]. Regarding energy consumption, while this study does not include a Life Cycle Assessment (LCA) to evaluate the CO_2_ footprint of this approach compared to classical esterifications, such an analysis would provide valuable insights into the overall sustainability of the process [64]. This aspect represents a potential avenue for future research to quantify the broader environmental benefits of enzymatic catalysis over traditional chemical methodologies. Overall, this enzymatic approach adheres to green chemistry principles by leveraging a highly selective biocatalyst, operating without hazardous reagents, and avoiding the harsh reaction conditions typically required in chemical esterifications. The findings of this study further support the viability of enzymatic synthesis as a sustainable alternative to conventional methodologies in biocatalysis-driven organic transformations.

## 4. Conclusions

Xylitol monoferulate was produced via biocatalyzed esterification with CaLB in a monophasic system, and the reaction conditions were optimized to achieve a maximum conversion yield of >98 ± 2%. The synthesized bifunctional prodrug, characterized here for the first time via NMR spectroscopy, represents a promising advancement in the enzymatic synthesis of bioactive derivatives with potential pharmacological applications. To further explore its prodrug behavior, future studies should focus on evaluating its hydrolytic stability and metabolic bioconversion in physiologically relevant environments. A key aspect to investigate is the hydrolysis rate of the ester bond, particularly in simulated gastric (SGFs) and intestinal fluids (SIFs), to assess whether the compound remains stable under gastrointestinal conditions or undergoes pre-systemic hydrolysis, influencing its oral bioavailability. Additionally, ex vivo assays using human liver homogenates (S9 fractions, microsomes) or plasma could provide crucial insights into its hepatic and systemic metabolism, clarifying whether hydrolysis by carboxylesterases or other metabolic enzymes facilitates a controlled release of ferulic acid in vivo. Beyond metabolism, further studies could explore the pharmacokinetic profile of xylitol monoferulate, including its plasma stability, absorption, and clearance, to determine its potential as a prodrug with improved bioavailability. Moreover, investigating its antioxidant, anti-inflammatory, and neuroprotective activities, both in vitro and in vivo, would help establish whether xylitol conjugation enhances the biological efficacy of ferulic acid while also providing prebiotic benefits. These studies would be fundamental in assessing whether xylitol monoferulate could serve as a novel biocompatible carrier for controlled ferulic acid release, offering both pharmacological and nutraceutical advantages.

## Figures and Tables

**Table 1:** The optimization of reaction conditions for the enzymatic esterification of ferulic acid (FA) with xylitol using *Candida antarctica* lipase B (CaLB) as a biocatalyst. The table summarizes the parameters tested, including variations in the temperature, enzyme concentration, the acid-to-alcohol molar ratio, the solvent volume, and the presence of molecular sieves, to determine the optimal conditions for selective monoester formation.

Parameter	Conditions Tested
Reaction vessel	The 10 mL screw-capped flasks in an oil bath with magnetic stirring
Substrates	Ferulic acid (FA, **1**) and xylitol (**2**)
Molar ratio (FA:xylitol)	1:1, 1:3, 1:5, 3:1, 5:1
Solvent	*t*-amyl alcohol
Solvent volume	2 mL, 5 mL
Temperature range	50 °C, 70 °C, 90 °C
Biocatalyst	*Candida antarctica* lipase B (CaLB), immobilized
Enzyme loading	10, 50, 100 g/L
Molecular sieves	0 mg, 100 mg
Reaction monitoring	¹H-NMR spectroscopy at 24 h, 48 h, 72 h, up to 192 h
Product formed	Xylitol monoferulate (XMF, **3**)
Byproduct	Traces of xylitol bis(ferulate) (**4**) detected at 1:1 molar ratio and 50 °C
Product purification	Flash column chromatography
Conversion calculation	Ratio of ester peak (**3**) (δ = 6.4 ppm) to the sum of ester (**3**) and acid (**1**) (δ = 6.3 ppm), multiplied by 100
Yield determination	If (**1**) is limiting: conversion = yield If (**2**) (xylitol) is limiting: yield calculated based on initial molar fraction of (**2**)

## Data Availability

The original contributions presented in this study are included in the article/supplementary material. Further inquiries can be directed to the corresponding author.

## Supplementary Materials

The following supporting information can be downloaded at https://www.mdpi.com/article/10.3390/biotech14020025/s1, Figure S1: ^1^H-NMR of xylitol monoferulate (**3**); Figure S2: ^13^C-NMR of xylitol monoferulate (**3**), Figure S3: gCOSY-NMR of xylitol monoferulate (**3**); Figure S4: gHSQC-NMR of xylitol monoferulate (**3**); Figure S5: gHMBC-NMR of xylitol monoferulate (**3**).

## Abbreviations

The following abbreviations are used in this manuscript:

FA	Ferulic acid
CaLB	*Candida antarctica* Lipase Type B
XMF	Xylitol monoferulate
