# Supplementary material for: Green Biocatalysis of Xylitol Monoferulate: Candida antarctica Lipase B-Mediated Synthesis and Characterization of Novel Bifunctional Prodrug
Source: BioTech (Basel). 2025 Apr 2;14(2):25. doi: 10.3390/biotech14020025 (PMC12015828; doi:10.3390/biotech14020025)
Supplement: Supplementary file 1 [file biotech-14-00025-s001.zip › biotech-3477919-supplementary.pdf]

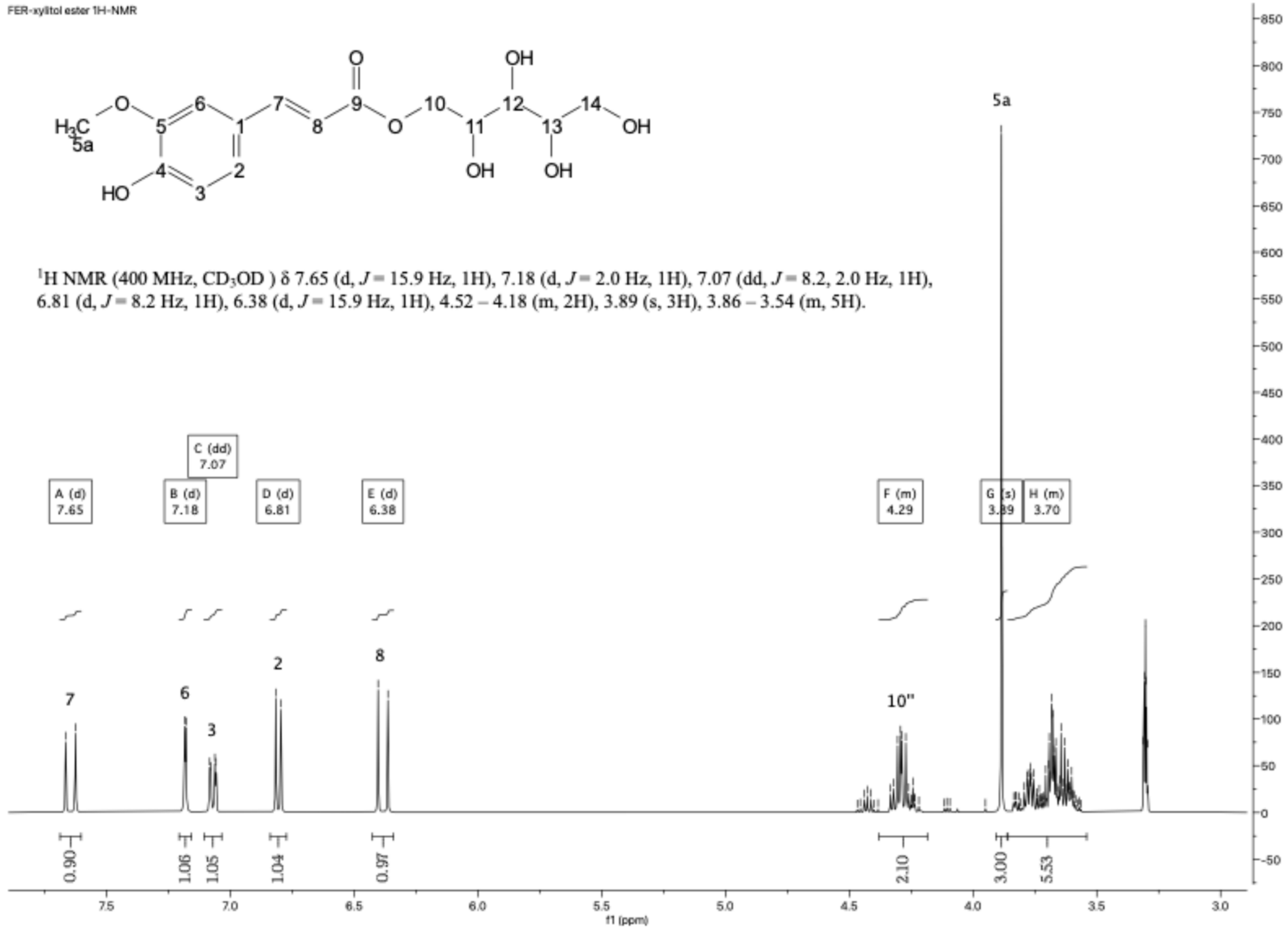

Supplementary Figure 1: ^{1}H-NMR of xylitol monoferulate (**3**)

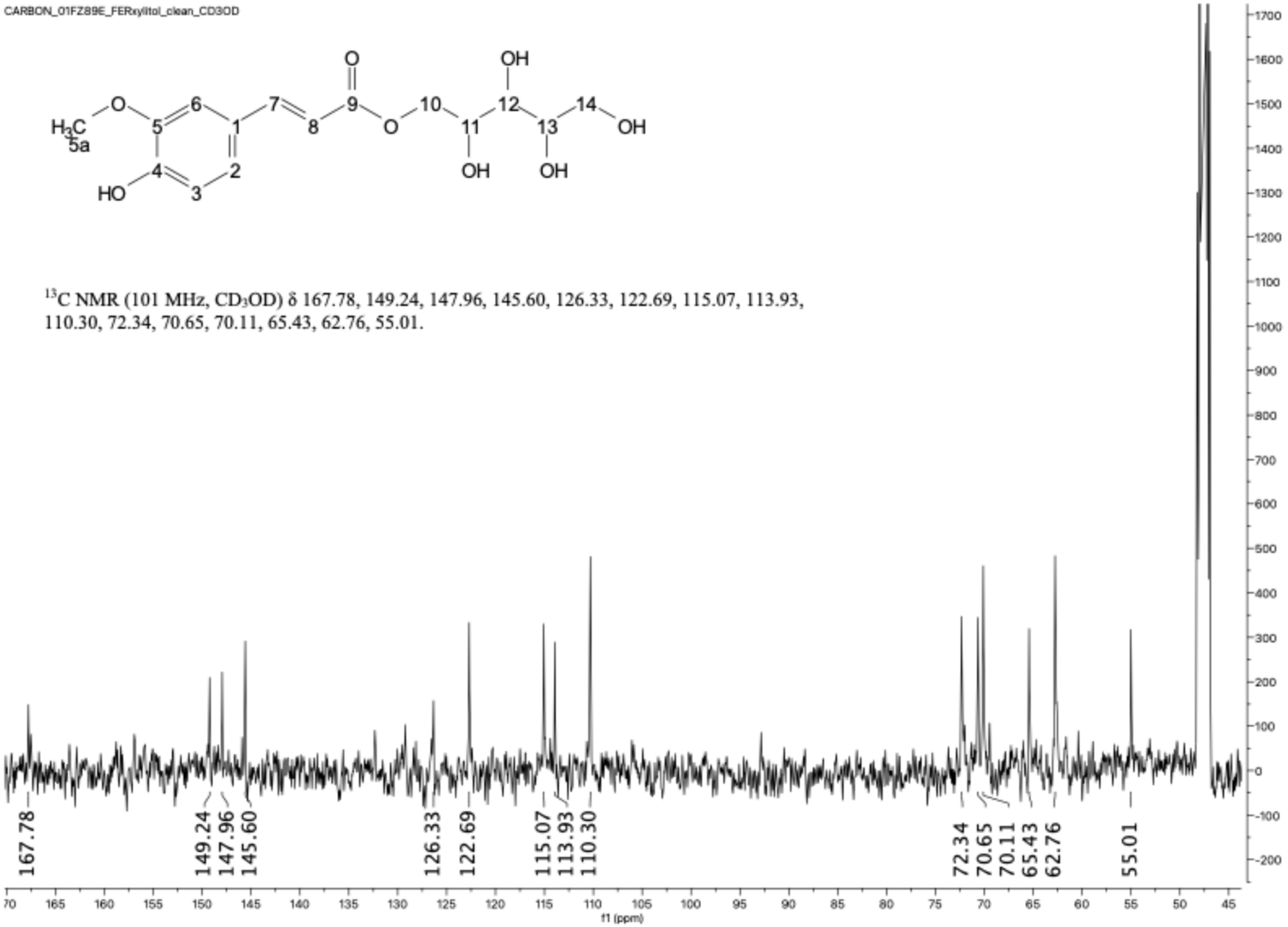

Supplementary Figure 2: ^{13}C-NMR of xylitol monoferulate (**3**)

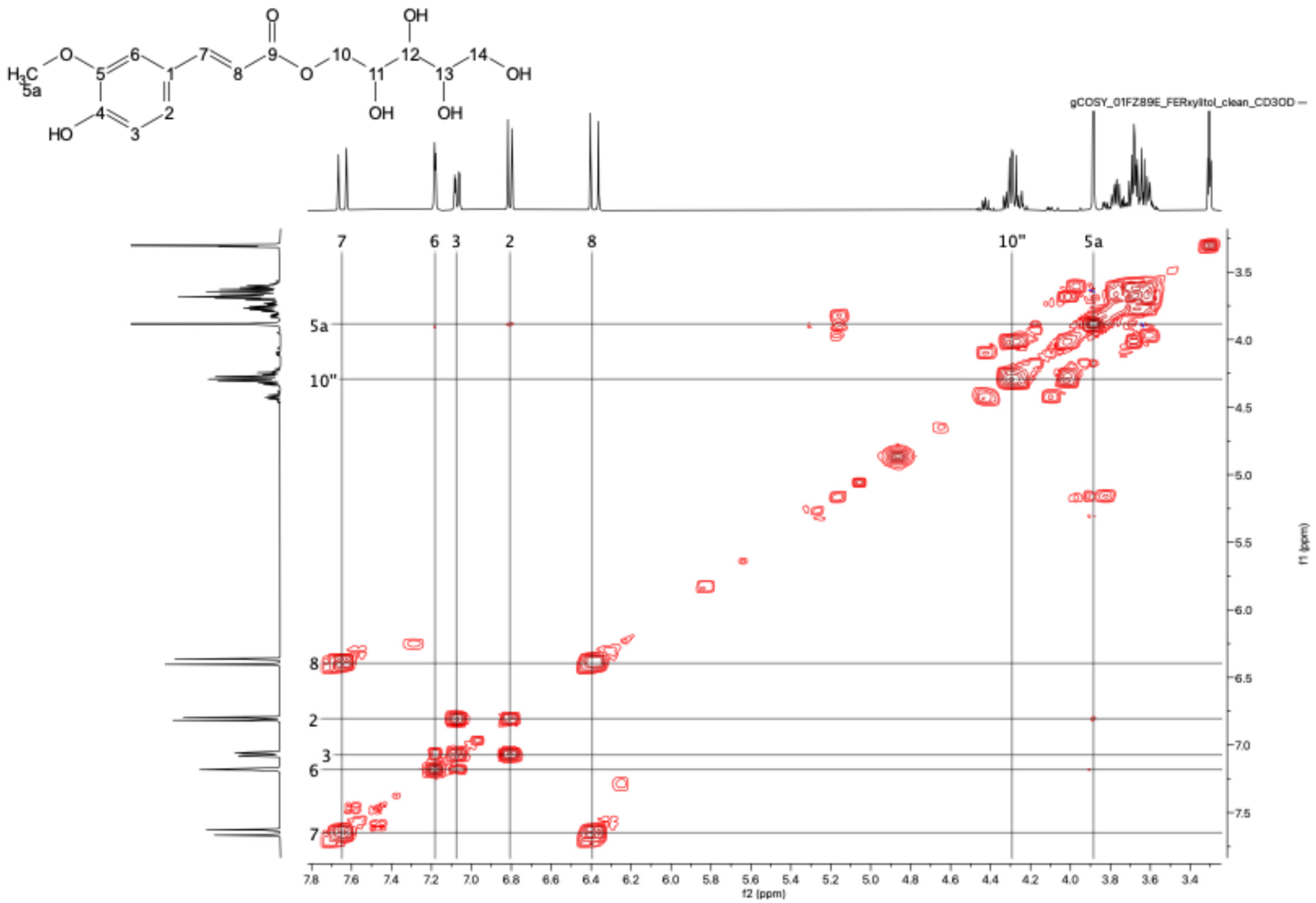

Supplementary Figure 3: gCOSY-NMR of xylitol monoferulate (**3**)

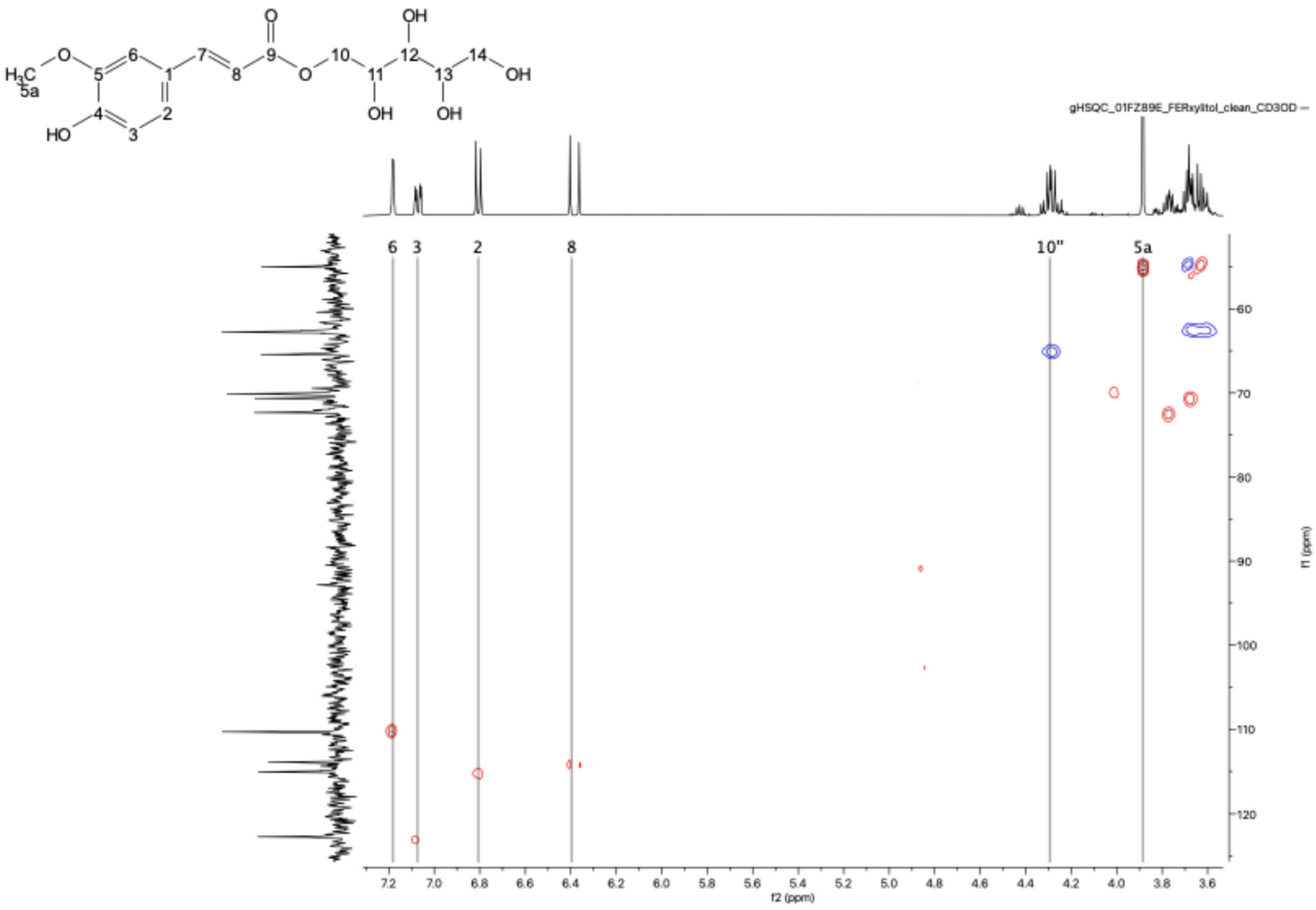

Supplementary Figure 4: gHSQC-NMR of xylitol monoferulate (**3**)

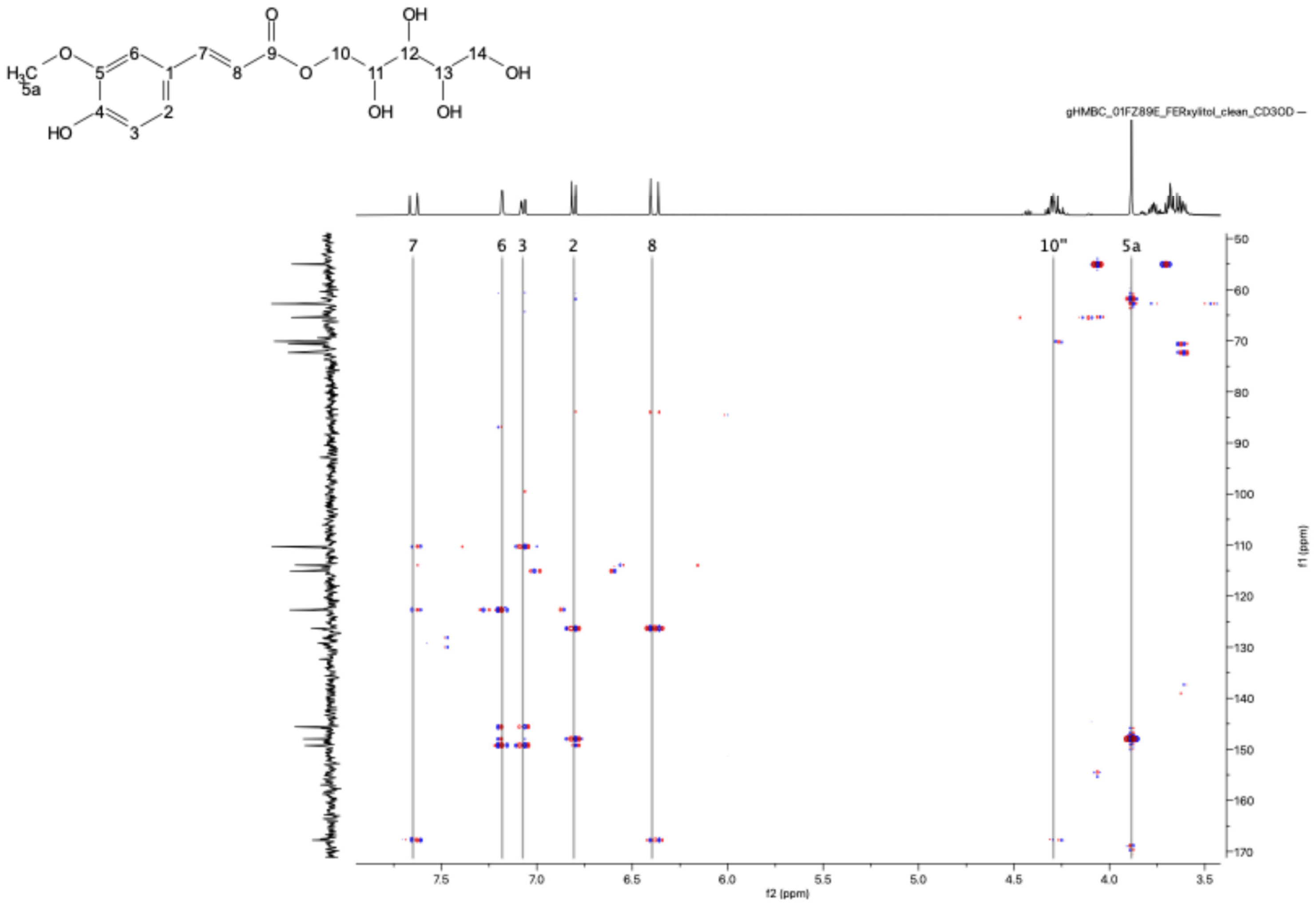

Supplementary Figure 5: gHMBC-NMR of xylitol monoferulate (**3**)
